# Head-to-Head Comparison of SSTR Antagonist [^68^Ga]Ga-DATA^5m^-LM4 with SSTR Agonist [^68^Ga]Ga-DOTANOC PET/CT in Patients with Well Differentiated Gastroenteropancreatic Neuroendocrine Tumors: A Prospective Imaging Study

**DOI:** 10.3390/ph17030275

**Published:** 2024-02-22

**Authors:** Rahul Viswanathan, Sanjana Ballal, Madhav P. Yadav, Frank Roesch, Parvind Sheokand, Swayamjeet Satapathy, Madhavi Tripathi, Shipra Agarwal, Euy Sung Moon, Chandrasekhar Bal

**Affiliations:** 1 Department of Nuclear Medicine, All India Institute of Medical Sciences, New Delhi 110029, India; rahulracks@gmail.com (R.V.); mail.sanjanaballal87@gmail.com (S.B.); madhav_yadav2000@yahoo.com (M.P.Y.);; 2 Department of Chemistry—TRIGA Site, Johannes Gutenberg University, 55128 Mainz, Germany; 3 Department of Pathology, All India Institute of Medical Sciences, New Delhi 110029, India

**Keywords:** neuroendocrine tumors, NETs, [^68^Ga]Ga-DATA^5m^-LM4, [^68^Ga]Ga-DOTANOC, PET/CT, SSTR antagonist

## Abstract

Neuroendocrine tumors (NETs) are slow-growing tumors that express high levels of somatostatin receptors (SSTRs). Recent studies have shown the superiority of radiolabeled SSTR antagonists in theranostics compared to agonists. In this prospective study, we compared the diagnostic efficacy between [^68^Ga]Ga-DOTANOC and [^68^Ga]Ga-DATA^5m^-LM4 in the detection of primary and metastatic lesions in patients with well differentiated gastroenteropancreatic (GEP) NETs. Histologically proven GEP-NET patients underwent [^68^Ga]Ga-DOTANOC & [^68^Ga]Ga-DATA^5m^-LM4 PET/CT scans, which were analyzed. The qualitative analysis involved the visual judgment of radiotracer uptake validated by the morphological findings using CT, which was considered as the reference standard. Quantitative comparisons were presented as the standardized uptake value (SUV) corrected for lean body mass: SULpeak, SULavg, and tumor-to-background ratios (TBR). In total, 490 lesions were confirmed via diagnostic CT. The lesion-based sensitivity of [^68^Ga]Ga-DATA^5m^-LM4 PET/CT was 94.28% (462/490) and 83.46% (409/490) for [^68^Ga]Ga-DOTANOC PET/CT (*p* < 0.0001). [^68^Ga]Ga-DATA^5m^-LM4 had statistical significance over [^68^Ga]Ga-DOTANOC in liver metastases [100% vs. 89.4%; *p* < 0.0001 (292 vs. 253 {283 lesions on CT})] and bone metastases [100% vs. 82.9%; *p* = 0.005 (45 vs. 34 {41 lesions on CT})]. Statistical significance was also noted for the TBR SULpeak of the primary and liver lesions. [^68^Ga]Ga-DATA^5m^-LM4 showed better sensitivity and a higher target-to-background ratio than [^68^Ga]Ga-DOTANOC PET/CT. [^68^Ga]Ga-DATA^5m^-LM4 PET/CT can be used to quantify the extent of skeletal and liver metastases for better planning of SSTR agonist- or antagonist-based therapy.

## 1. Introduction

Gastroenteropancreatic neuroendocrine tumors (GEP-NETs) are slow-growing tumors characterized by high levels of somatostatin receptors (SSTRs)—notably subtypes 1 and 2 (SSTR1 and SSTR2) [1]. Somatostatin receptor (SSTR) imaging utilizes radiolabeled somatostatin analogs that bind to the somatostatin receptors (SSTR1–5), commonly found in abundance in neuroendocrine tumors (NETs). By assessing the SSTR status in NETs in vivo, this imaging method also determines patients’ eligibility for peptide receptor radionuclide therapy (PRRT), which is an effective and safe treatment approach for advanced or progressing SSTR-positive NETs [2,3].

The pharmacomodulation of synthetic somatostatin analogs has resulted in changes to the chirality of the first amino acid (from D to L form) and cysteine number 2 (from L to D form), giving rise to a novel class of SSTR-specific compounds with antagonist effects. From a pharmacological standpoint, the biological and molecular mechanisms responsible for their targeting efficacy in vivo differ significantly. While agonist analogs are internalized into the cell as ligand-receptor complexes upon binding to a somatostatin receptor (SSTR), this internalization does not occur, or occurs minimally, for somatostatin antagonists. Antagonists do not stimulate the G-protein coupled to the SSTR, thus blocking agonist-induced activity. Interestingly, it has been demonstrated that the imaging of transmembrane receptors can still be effective without the internalization of the ligand-receptor complex. In some cases, antagonist analogs may even exhibit superior behavior to agonists, such as enhanced accumulation in tumor tissue, reduced kidney retention, and rapid clearance [4]. This increased tumor uptake may result from a greater number of target binding sites available for antagonists and slower dissociation rates compared to agonists, allowing for prolonged accumulation of the radiotracer at the tumor tissue [5,6]. Ginj et al. [7] proposed a paradigm shift, suggesting that radiolabeled SSTR antagonists may perform better than agonists despite the absence of internalization.

Commonly used gallium-68 radiopharmaceuticals are based on the octadentate bifunctional chelator-DOTA (DOTA = tetraazacyclododecane-1,4,7,10-tetraacetate). DOTA-based precursors are a requirement for relatively harsh conditions (heating at 95 °C for 10 to 30 min at a pH of 4.6) for radiolabeling. This is because of mismatch between the small ionic radius of Ga(III) and the large cavity size of the tetraaza-macrocyclic DOTA, which fits larger metal ions (i.e., yttrium, the lanthanide ions, and calcium) more efficiently.

DATA chelators are a novel class of tri-anionic ligands, based on 6-amino-1,4-diazepine-triacetic acid, which have a smaller cavity size better suited for the chelation of Gallium-68. Moreover, they represent a novel class of hybrid chelators due to the one nitrogen atom arranged in exo-position. The remaining cyclic moiety is supposed to maintain high kinetic stability in vivo, while the acyclic moiety guarantees easy labelling. [^68^Ga]Ga-DATA conjugated radiopharmaceuticals can be produced at room temperature within a short time at >95% yield that does not require post-labelling purification to meet pharmacopeia standards [8]. The hexadentate DATA^m^ ligand and its bifunctional analogue, DATA^5m^, rapidly form complexes with Gallium-68 in high radiochemical yield. The stability constants of DATA^m^ and DATA^5m^ complexes formed with Ga^3+^, Zn^2+^, Cu^2+^, Mn^2+^, and Ca^2+^ have been determined by pH-potentiometry, spectrophotometry (Cu^2+^), and 1H- and 71Ga-NMR spectroscopy (Ga^3+^) [9].

**Figure 1:**
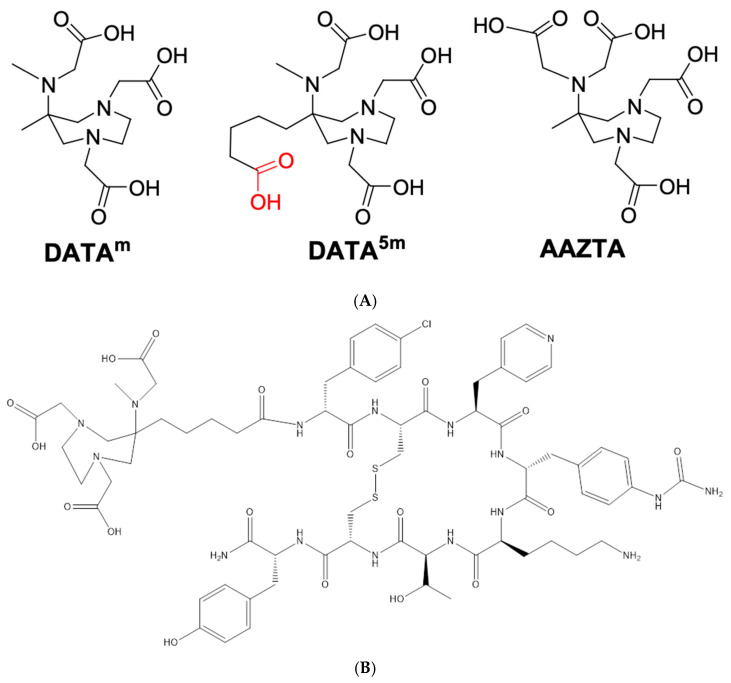
(**A**) Structure of DATA^m^, DATA^5m^, and AAZTA. (**B**) Structure of DATA^5m^-LM4.

In parallel, the AAZTA heptadentate ligand (Figure 1A) can be readily synthesized. Its coordination properties with a wide range of metal ions, including lanthanides and transition metals, have been well-documented. Recent advancements have focused on its lipophilic derivatives for targeting high-density lipoproteins (HDL), cell membranes, and the synthesis of bifunctional compounds for conjugation purposes. The rapid formation of complexes using AAZTA has inspired research into its potential for developing useful complexes for targeted PET applications [10,11].

Moreover, the H3DATA^m^ and H4DATA^5m^ ligands, derived from H4AAZTA, feature a substitution of one of the carboxylate groups with a methyl group in the imino-diacetate (IMDA) moiety [12]. Notably, the stability constants of Ga(DATA^m^) and Ga(DATA^5m^) complexes are slightly higher than those of Ga(AAZTA) [13]. This enhanced stability underscores their potential for various applications, particularly in targeted PET imaging. A comprehensive study involving the evaluation of three different SSTR2 antagonists, namely LM3, JR10 (p = NO_2_-Phe-c[-Cys-Tyr-Aph(Cbm)-Lys-Thr-Cys]-Tyr-NH_2_), and JR11 (Cpa-c[-Cys-Aph(Hor)--Aph(Cbm)-Lys-Thr-Cys]--Tyr-NH_2_), in combination with two chelators (DOTA and NODAGA) and various (radio)metals, including In(III), Y(III), Lu(III), Cu(II), and Ga(III), clearly demonstrated the extremely high sensitivity of the antagonists [14]. Leung et al. stated that JR11 is a novel SSTR2 antagonist with promising results in preclinical studies, but the clinical evidence is scarce [15]. Antagonists, such as [^68^Ga]Ga-NODAGA-JR11 and [^68^Ga]Ga-DOTA-JR11, demonstrated superior detection efficacy compared to agonists like [^68^Ga]Ga-DOTATATE in patients with metastatic, well-differentiated neuroendocrine tumors. These findings underscore the potential advantages of utilizing antagonists for improved sensitivity, lesion detection, and image contrast in this patient population [16,17,18].

The new tracer DATA^5m^-LM4 is based on a known SSTR2 antagonist LM3 [14,19] following 4Pal^3^/Tyr^3^-substitution in the cyclic octapeptide chain [20]. Notably, DATA^5m^-LM4 can be labeled with Ga-67/68 at much lower temperatures than other DOTA-derivatized peptides (including DOTA-LM3) by virtue of the hybrid DATA^5m^ chelator attached on its N-terminus. The precursor DATA^5m^-LM4, developed for the Gallium-68 labeling of somatostatin receptor 2 (SSTR2), consists of the DATA^5m^ chelate. It is covalently bound to the SSTR2 ligand, -LM4 = Cpa-cyclo[DCys-Pal-Daph(Cbm)-Lys-Thr-Cys]Dtyr-NH_2_ where Cpa represents 4-chlorophenylalanine and Pal indicates pyridylalanine. The molecule is illustrated in Figure 1B.

Given the promising preclinical data and initial clinical findings, along with the scarcity of comprehensive clinical reports on the radiolabelled SSTR-antagonist [^68^Ga]Ga-DATA^5m^-LM4, our objective is to conduct a systematic prospective study. The goal of the study is to compare diagnostic efficacy between the SSTR-antagonist [^68^Ga]Ga-DATA^5m^-LM4 with those obtained using the approved somatostatin receptor agonistic imaging agent, [^68^Ga]Ga-DOTANOC, in patients diagnosed with histopathologically confirmed gastroenteropancreatic neuroendocrine tumors (GEP-NETs).

## 2. Results

Out of the 54 patients who underwent scans, 50 individuals diagnosed with gastroenteropancreatic neuroendocrine tumors (24 males and 26 females) with a mean age of 44.7 ± 13.7 years (range: 14 to 71) met the eligibility criteria and were included in the study analysis. Following an intravenous injection of [^68^Ga]Ga-DATA^5m^-LM4, all vital signs remained within normal limits, and no adverse events were observed. Among the 50 patients, 33 had received prior treatments. The median serum chromogranin-A level at the time of imaging was 201 ng/mL (25th–75th percentile: 94 to 812 ng/mL). A total of 13 patients underwent surgical interventions (26%), 14 patients (26%) had received octreotide injections at least one month prior, 3 patients were treated with mTOR inhibitors (6%), 1 patient was on the CAPTEM regimen (2%), and 3 patients underwent [^177^Lu]Lu-DOTATATE therapy (6%). Patient demographics are described in Table 1. Detailed patient clinical parameters are provided in Table A1 in Appendix A.

### 2.1. Biodistribution of [^68^Ga]Ga-DATA^5m^-LM4

**Figure 2:**
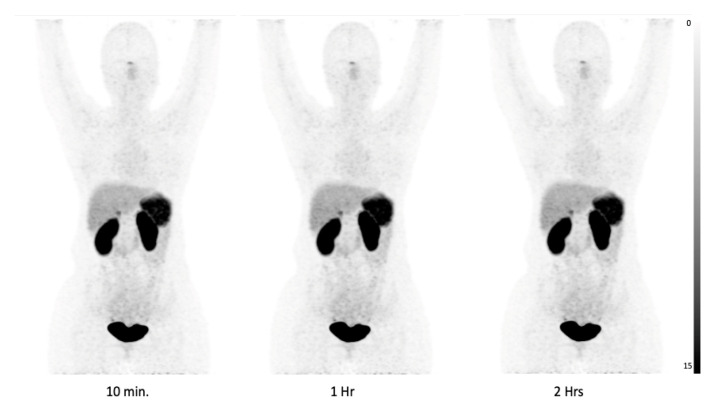
Serial time point imaging in patient 27, a 35-year-old female, k/c/o Duodenal NET post-surgery. Images were taken at 10 min, 1 h and 2 h time intervals from the time of injection of [^68^Ga]Ga-DATA^5m^-LM4. Theses MIP images depict the normal biodistribution of the tracer at spleen, liver, and kidney and minimal uptake in the pituitary and salivary glands.

**Figure 3:**
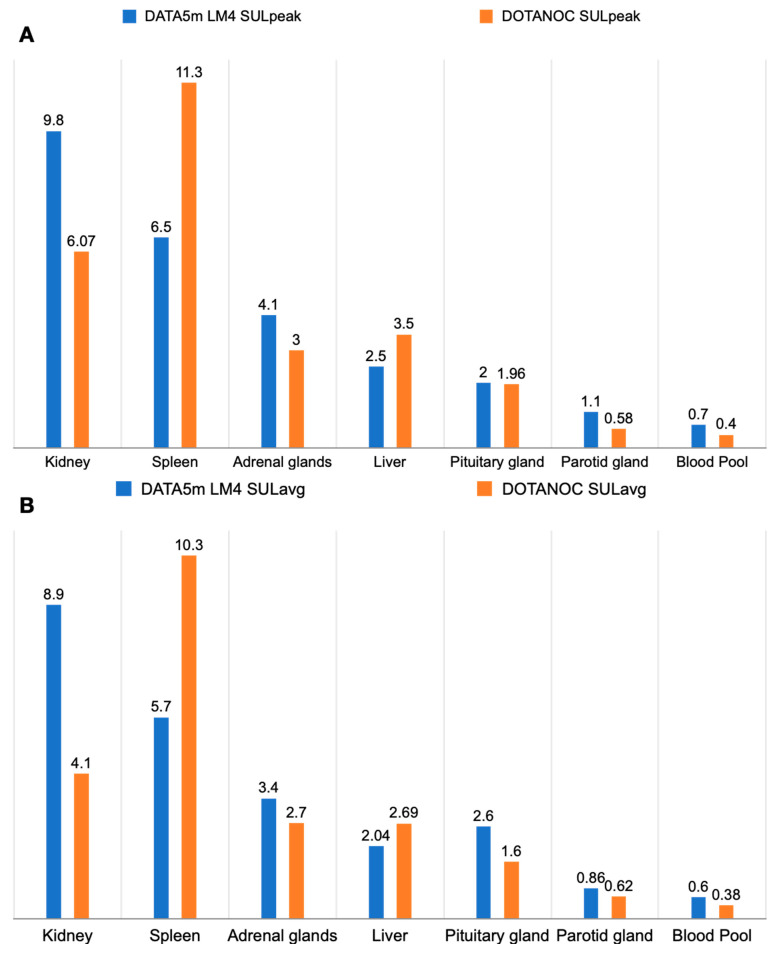
(**A**) Comparison of SULpeak between [^68^Ga]Ga-DATA^5m^-LM4 and [^68^Ga]Ga-DOTANOC for physiological organs. (**B**) Comparison of SULavg between [^68^Ga]Ga-DATA^5m^-LM4 and [^68^Ga]Ga-DOTANOC for physiological organs.

Figure 2 illustrates the biodistribution pattern of [^68^Ga]Ga-DATA^5m^-LM4 in one of the patients. The radiotracer displayed rapid clearance from the blood circulation, primarily excreting through the kidneys and urinary tract. Among normal organs, the highest accumulation of [^68^Ga]Ga-DATA^5m^-LM4, besides the kidneys {9.8; (6.6 to 13.8)}, was observed in the spleen {6.6; (4.0 to 9.7)} and adrenal glands {4.1; (2.4 to 6.1)}, followed by the liver {2.5; (1.7 to 3.8)} and pituitary {2.1; (1.2 to 3.5)}. Data are presented as SULpeak Median (IQR) at 60 min post-injection (refer to Table 2). In contrast, uptake in the skeletal system, brain, lungs, blood pool, mediastinum, bone marrow, and muscle remained at background levels. Figure 3A,B depict the SULpeak and SULavg of both [^68^Ga]Ga-DATA^5m^-LM4 and [^68^Ga]Ga-DOTANOC, respectively, illustrating their biodistribution in physiological organs.

### 2.2. Comparison of Lesion Detection and Uptake between [68Ga]Ga-DOTANOC and [68Ga]Ga-DATA5m-LM4

#### 2.2.1. Primary Tumor

**Figure 4:**
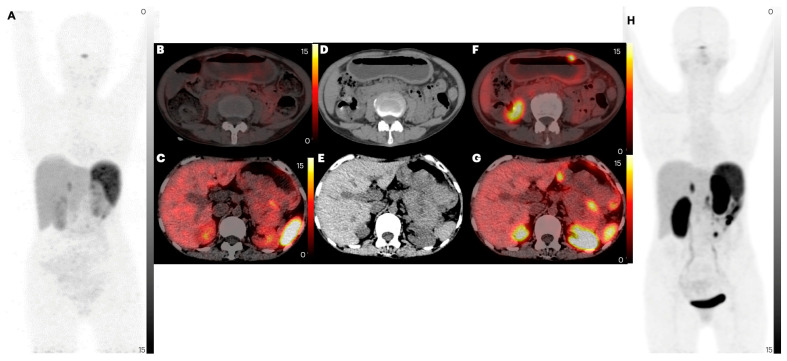
PET/CT images of patient number 17, a 44-year-old female with a known case of NET stomach grade 3, for whom on-table swallowing of water was undertaken for dilatation of stomach for clear visualization of the lesions. Images (**A**,**H**) are MIP images of [^68^Ga]Ga-DOTANOC and [^68^Ga]Ga-DATA^5m^-LM4 PET/CT, respectively. Images (**B**,**C**) are [^68^Ga]Ga-DOTANOC PET/CT axial images. Images (**D**,**E**) are CT axial images. Images (**F**,**G**) are [^68^Ga]Ga-DATA^5m^-LM4 PET/CT axial images. Increased number of gastric (primary) foci of tracer uptake are noted in [^68^Ga]Ga-DATA^5m^-LM4 PET/CT images.

**Figure 5:**
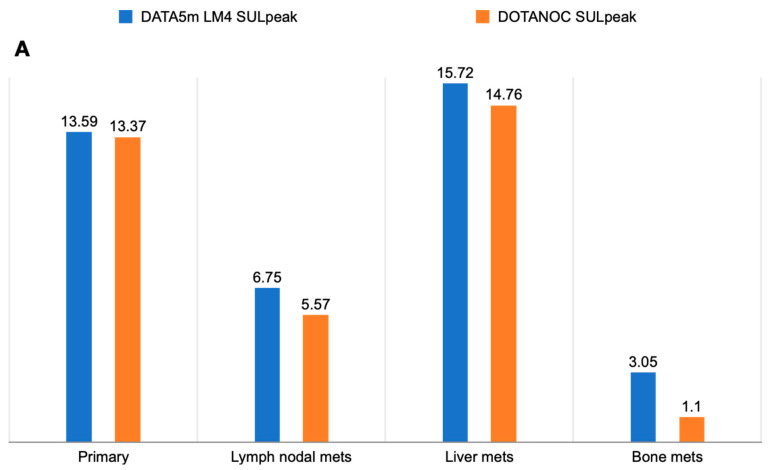

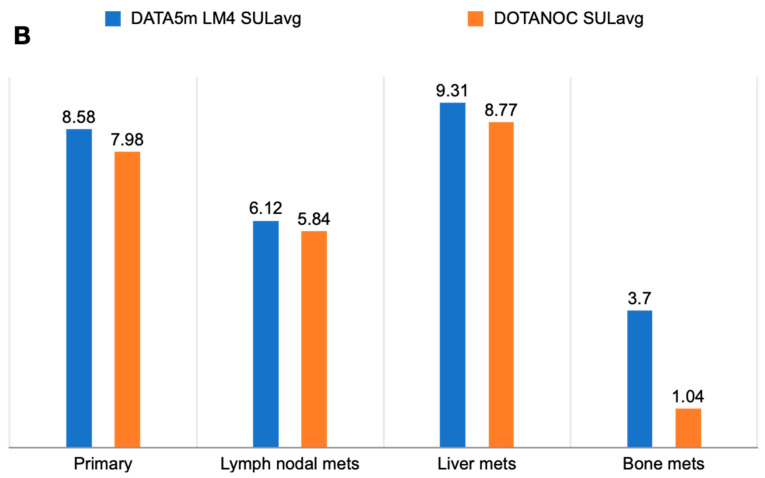
(**A**) Comparison of SULpeak between [^68^Ga]Ga-DATA^5m^-LM4 and [^68^Ga]Ga-DOTANOC in primary and metastatic lesions. (**B**) Comparison of SULavg between [^68^Ga]Ga-DATA^5m^-LM4 and [^68^Ga]Ga-DOTANOC in primary and metastatic lesions.

**Figure 6:**
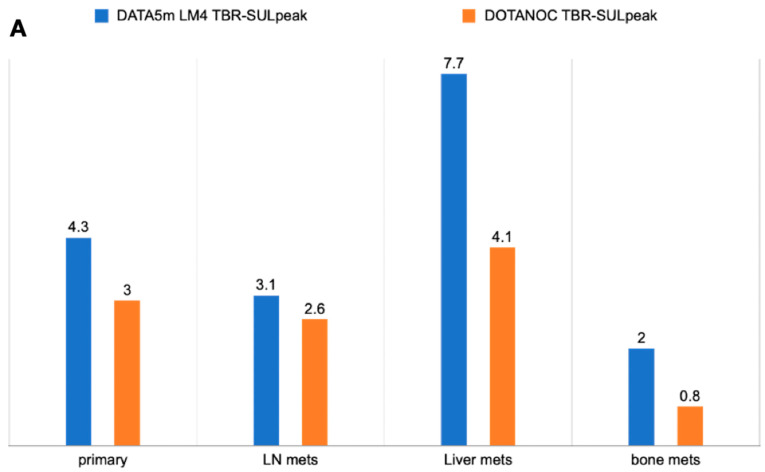

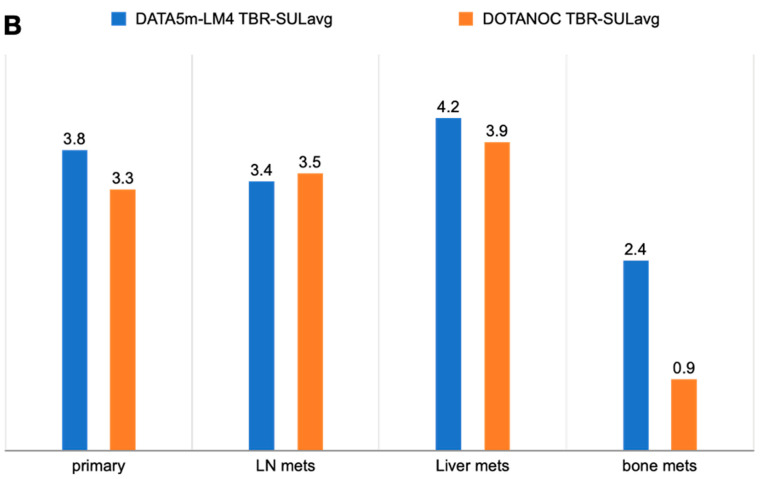
(**A**) TBR SULpeak of [^68^Ga]Ga-DATA^5m^-LM4 and [^68^Ga]Ga-DOTANOC. (**B**) TBR SULavg of [^68^Ga]Ga-DATA^5m^-LM4 and [^68^Ga]Ga-DOTANOC.

Among the 50 patients, 47 primary lesions were identified on diagnostic CT scans. An intense uptake of [^68^Ga]Ga-DATA^5m^-LM4 was observed in 42 lesions (89.36%), while [^68^Ga]Ga-DOTANOC detected expression in 40 lesions (85.10%) (*p* = 0.544) (Figure 4). The median SULpeak values were similar between the antagonist and agonist tracers {SULpeak; [^68^Ga]Ga-DATA^5m^-LM4: 13.6 (IQR: 6.6 to 20.2) vs. [^68^Ga]Ga-DOTANOC: 13.4 (IQR: 6.1 to 19.0, *p* = 0.316)} (refer to Table 3) (Figure 5A,B). However, the TBR SULpeak was significantly higher with [^68^Ga]Ga-DATA^5m^-LM4 compared to [^68^Ga]Ga-DOTANOC {SULpeak; [^68^Ga]Ga-DATA^5m^-LM4: 4.3 (IQR: 3.1 to 8.4) vs. [^68^Ga]Ga-DOTANOC: 3.0 (IQR: 2.0 to 5.6); *p* = 0.014} (Figure 6A,B).

#### 2.2.2. Lymph Node Metastases

**Figure 7:**
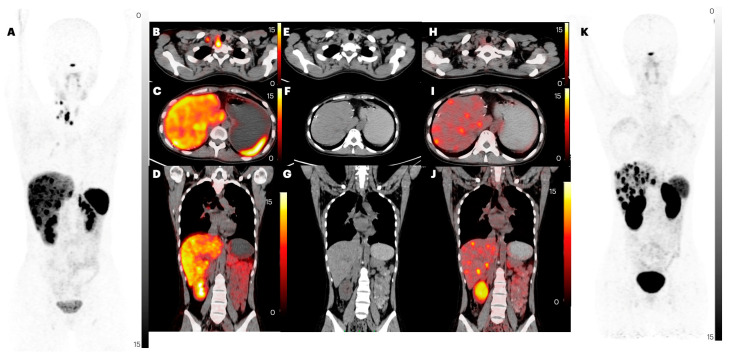
PET/CT images of patient number 10, a 25-year-old female with a known case of small intestine NET with liver and neck lymph nodal metastasis, post-surgical resection and anastomosis of small intestine with left hepatectomy, Injection octreotide, and [^177^Lu]Lu-DOTATATE. Images (**A**,**K**) are MIP images of [^68^Ga]Ga-DOTANOC and [^68^Ga]Ga-DATA^5m^-LM4 PET/CT, respectively. Images (**B**,**C**) are axial images of [^68^Ga]Ga-DOTANOC. (**E**,**F**) are axial images of CT, and (**H**,**I**) are axial images of [^68^Ga]Ga-DATA^5m^-LM4 PET/CT, showing positive neck nodes in (**B**) that are not noted in (**H**). Images (**C**,**F**,**I**) are axial sections of [^68^Ga]Ga-DOTANOC, CT, and [^68^Ga]Ga-DATA^5m^-LM4 PET/CT showing liver metastases. Images (**D**,**G**,**J**) are coronal section images of [^68^Ga]Ga-DOTANOC, CT, and [^68^Ga]Ga-DATA^5m^-LM4 PET/CT, respectively, showing liver metastases. In the images (**A**,**B**,**E**,**H**,**K**), lymph node uptake is observed in the scans using [^68^Ga]Ga-DOTANOC. However, in the corresponding [^68^Ga]Ga-DATA^5m^-LM4 PET/CT images, there is no detectable lymph node uptake. When examining images (**A**,**C**,**F**,**D**,**G**,**J**,**K**), an equivalent number of liver lesions are observed in both [^68^Ga]Ga-DOTANOC and [^68^Ga]Ga-DATA^5m^-LM4 PET/CT scans. However, the crucial distinction lies in the contrast quality: [^68^Ga]Ga-DOTANOC exhibits a lower TBR (target-to-background ratio), whereas [^68^Ga]Ga-DATA^5m^-LM4 PET/CT images demonstrate a higher TBR. This disparity signifies superior image contrast in the latter, highlighting its potential for more accurate and detailed imaging.

Out of the 50 patients, 31 had a total of 107 lymph node (LN) metastases identified on CT scans. While [^68^Ga]Ga-DATA^5m^-LM4 accurately diagnosed 77.57% (83 out of 107) of LN metastases, [^68^Ga]Ga-DOTANOC diagnosed 76.63% (82 out of 107) (*p* = 0.862) (Figure 7). However, the SULpeak and TBR values of the lymph nodes were significantly higher with [^68^Ga]Ga-DATA^5m^-LM4 {SULpeak; [^68^Ga]Ga-DATA^5m^-LM4: 6.7 (IQR: 3.9 to 19.3) vs. [^68^Ga]Ga-DOTANOC: 5.6 (IQR: 3.3 to 20.5; *p* = 0.708} {TBR SULpeak; 3.1 (IQR: 1.5 to 6.1) vs. 2.6 (IQR: 0.7 to 5.3); *p* = 0.757} (Figure 6A,B).

#### 2.2.3. Lung Metastases

Lung metastases were observed on CT scans in eight patients. However, neither [^68^Ga]Ga-DATA^5m^-LM4 nor [^68^Ga]Ga-DOTANOC PET/CT images exhibited tracer uptake in the lung lesions. Upon a detailed lesion-based analysis, out of the right lung nodules identified on CT, neither [^68^Ga]Ga-DATA^5m^-LM4 nor [^68^Ga]Ga-DOTANOC PET/CT detected any tracer-avid lesions.

#### 2.2.4. Liver Metastases

**Figure 8:**
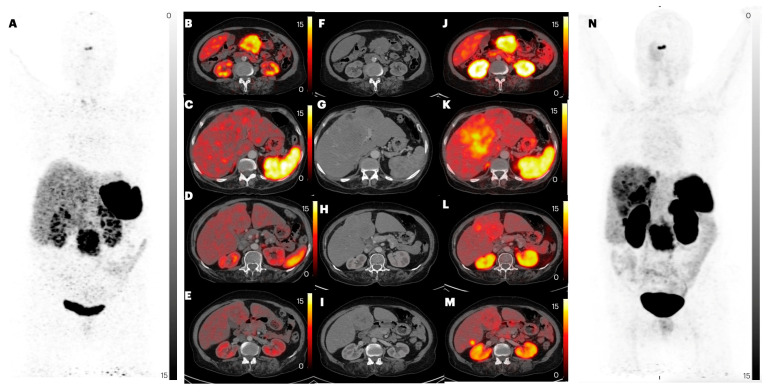
PET/CT images of patient number 16, a 64-year-old female with a known case of gastric NET with liver metastases with no treatment started and for whom IV contrast was given. Images (**A**,**N**) are MIP images of [^68^Ga]Ga-DOTANOC and [^68^Ga]Ga-DATA^5m^-LM4 PET/CT, respectively. Images (**B**,**C**,**D**,**E**) are [^68^Ga]Ga-DOTANOC PET/CT axial images. Images (**F**,**G**,**H**,**I**) are CT images. Images (**J**,**K**,**L**,**M**) are [^68^Ga]Ga-DATA^5m^-LM4 PET/CT images. Liver metastases with tracer uptake are noted in [^68^Ga]Ga-DATA^5m^-LM4 PET/CT images.

Out of the 50 patients, 31 (62%) were identified with liver metastasis on CT imaging, with [^68^Ga]Ga-DATA^5m^-LM4 demonstrating concordant findings in 28 out of 31 patients (90.3%). In comparison, [^68^Ga]Ga-DOTANOC detected liver metastases in 26 out of 31 patients (83.9%) (*p* = 0.449) (Figure 8). [^68^Ga]Ga-DATA^5m^-LM4 accurately diagnosed 292 out of 283 liver lesions (100%), whereas [^68^Ga]Ga-DOTANOC detected 89.4% (253 out of 283) of liver lesions (*p* < 0.0001). The SULpeak values of liver lesions were higher with [^68^Ga]Ga-DATA^5m^-LM4 compared to [^68^Ga]Ga-DOTANOC {SULpeak; [^68^Ga]Ga-DATA^5m^-LM4: 15.7 (IQR: 8.3 to 25.7) vs. [^68^Ga]Ga-DOTANOC: 14.7 (IQR: 7.3 to 20.9; *p* = 0.484} (refer to Table 3) (Figure 5A,B). Similarly, the TBR SULpeak values (i.e., liver lesion-to-healthy liver tissue ratios) were significantly higher (*p* = 0.008) with [^68^Ga]Ga-DATA^5m^-LM4: 7.71 (IQR: 3.3 to 14.4) compared to [^68^Ga]Ga-DOTANOC: 4.1 (IQR: 1.9 to 6.4) (Figure 6A,B).

#### 2.2.5. Bone Metastases

**Figure 9:**
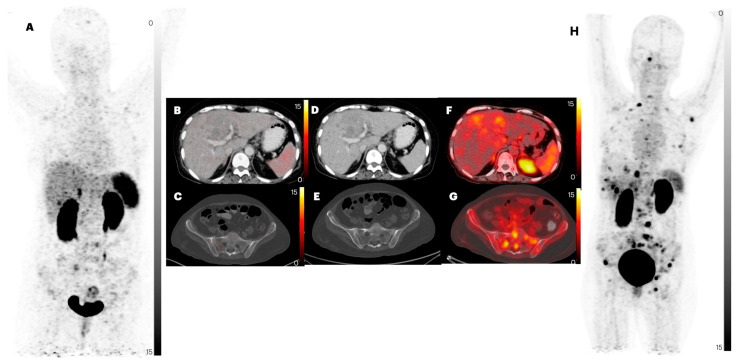
PET/CT images of patient number 2, a 50-year-old female with a known case of gallbladder NET with liver and skeletal metastases, post six cycles of chemotherapy and for whom IV contrast was given. Images (**A**,**H**) are MIP images of [^68^Ga]Ga-DOTANOC and [^68^Ga]Ga-DATA^5m^-LM4 PET/CT, respectively, with image (**H**) showing increased skeletal and liver metastases that are not noted in image (**A**). Images (**B**,**C**) are axial images of [^68^Ga]Ga-DOTANOC. (**D**,**E**) are axial images of CT, and (**F**,**G**) are axial images of [^68^Ga]Ga-DATA^5m^-LM4 PET/CT, showing liver and skeletal metastases that are not noted in (**B**,**C**).

Bone lesions were detected in 6 out of 50 patients (12%) (Table 3). Among these six patients, bone metastases were identified in all cases (100%) using [^68^Ga]Ga-DATA^5m^-LM4, as well as in all cases (100%) using [^68^Ga]Ga-DOTANOC PET/CT (*p* = 1.000). [^68^Ga]Ga-DATA^5m^-LM4 revealed a higher number of lesions compared to both CT (41 lesions) and [^68^Ga]Ga-DOTANOC {Number of bone lesions; [^68^Ga]Ga-DATA^5m^-LM4: 45 (100%) vs. [^68^Ga]Ga-DOTANOC: 34 (82.9%, *p* = 0.005} (Table 3 and Figure 9). The SULpeak values for bone lesions were 3.0 (IQR: 2.3 to 4.5) with [^68^Ga]Ga-DATA^5m^-LM4 and 1.1 (IQR: 1.0 to 1.6) with [^68^Ga]Ga-DOTANOC (*p* = 0.312) (Figure 5A,B). However, TBR values were significantly higher with [^68^Ga]Ga-DATA^5m^-LM4 compared to [^68^Ga]Ga-DOTANOC {SULpeak; [^68^Ga]Ga-DATA^5m^-LM4: 2.0 (IQR: 0.6 to 2.8) vs. [^68^Ga]Ga-DOTANOC: 0.8 (IQR: 0.3 to 1.6); *p* = 0.093} (Table 3 and Figure 6A,B).

#### 2.2.6. Brain Metastases

No metastatic brain lesions was found in CT, c or [^68^Ga]Ga-DOTANOC PET/CT.

#### 2.2.7. Other Distant Metastases

No other distant metastases was found in CT, [^68^Ga]Ga-DATA^5m^-LM4 PET/CT, or [^68^Ga]Ga-DOTANOC PET/CT.

#### 2.2.8. Effect of WHO Grade on SUL Uptake Values

**Figure 10:**
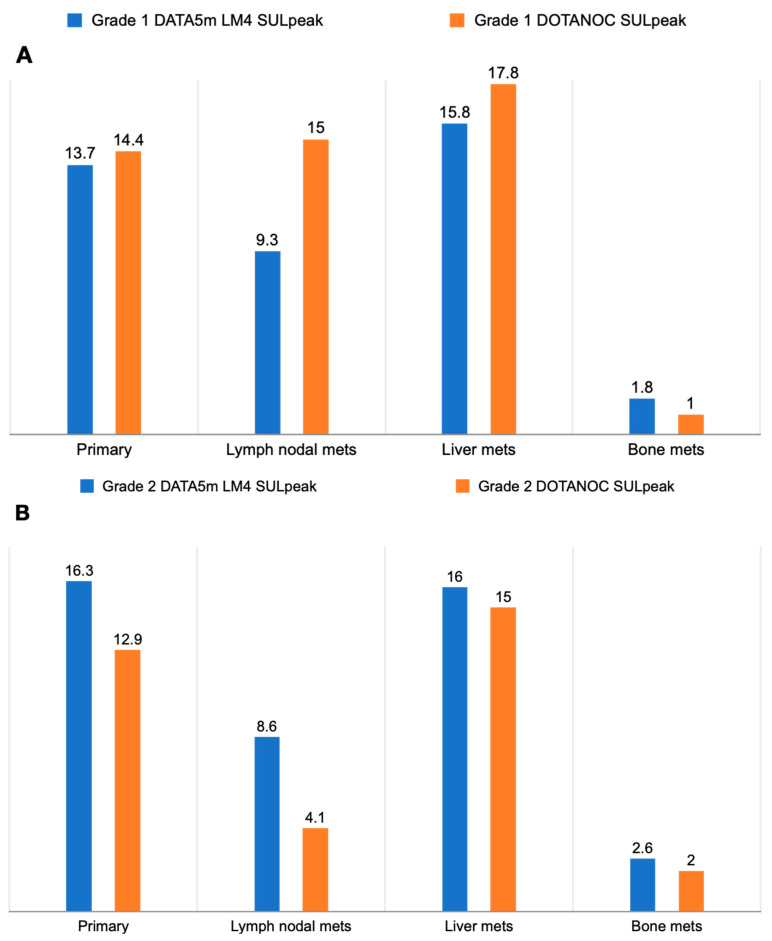

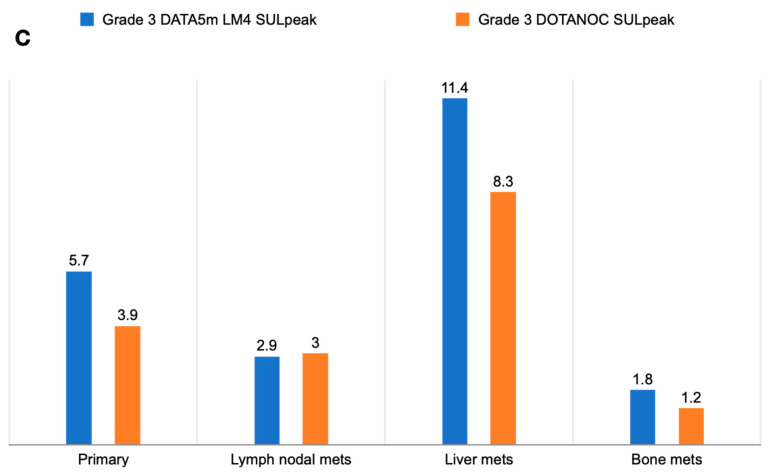
SULpeak values for primary and metastatic lesions of all the patients based on WHO grades of the patient. (**A**) WHO Grade I, (**B**) WHO Grade II, and (**C**) WHO Grade III.

Comparison of SULpeak values between [^68^Ga]Ga-DOTANOC and [^68^Ga]Ga-DATA^5m^-LM4 PET/CT scans revealed intriguing trends across different lesion grades (Figure 10). In grade I lesions, while SULpeak values increased with [^68^Ga]Ga-DOTANOC compared to [^68^Ga]Ga-DATA^5m^-LM4 in primary lymph nodal metastases and liver metastases, the reverse was observed in bone metastases, albeit not reaching statistical significance. However, in grade II and grade III lesions, SULpeak values of [^68^Ga]Ga-DATA^5m^-LM4 PET/CT scans exhibited higher values across all lesion types compared to [^68^Ga]Ga-DOTANOC scans, although these were again not statistically significant.

## 3. Discussion

### 3.1. Discussion of Current Study

[^67^Ga]Ga-DATA^5m^-LM4 was assessed in HEK293-SST2R cells and mouse models and directly compared with [^67^Ga]Ga-DOTA LM3 in a direct comparative analysis. The analysis of biodistribution in male SCID mice with twin HEK293-SSTR2 and wtHEK293 xenografts revealed extended tumor retention and reduced kidney uptake for [^67^Ga]Ga-DATA^5m^-LM4 in HEK293-SST2R xenograft-bearing mice, as opposed to [^67^Ga]Ga-DOTA LM3. In vivo stability was confirmed, with [^67^Ga]Ga-DATA^5m^-LM4 showing notably higher uptake in HEK293-SST2R cells. Remarkably, [^67^Ga]Ga-DATA^5m^-LM4 maintained a consistently elevated uptake in HEK293-SSTR2 tumors between 1 and 4 h post-injection, contrasting with the declining trend observed with [^67^Ga]Ga-DOTA LM3 during this period. Subsequent PET/CT imaging in a neuroendocrine tumor (NET) patient validated the strong SSTR2 binding affinity of [^68^Ga]Ga-DATA^5m^-LM4, aligning with findings from the mouse studies [21].

Through the modification of the SST2R-antagonist LM4 with the hybrid chelator AAZTA5, it becomes possible to label it with In-111 for diagnostic SPECT/CT and Lu-177 for radionuclide therapy, broadening its potential applications beyond [^68^Ga]Ga-DATA^5m^-LM4 PET/CT. The hybrid nature of DATA^5m^/AAZTA5 chelators enables quicker coordination of radiometals under gentler conditions, particularly beneficial in clinical environments. Consequently, preclinical evaluations of [^111^In]In-AAZTA5-LM4 and [^177^Lu]Lu-AAZTA5-LM4 were conducted, showcasing their exceptional qualities compared to the respective [^111^In]In-DOTA-LM3 and [^177^Lu]Lu-DOTA-LM3 references [22,23,24]. These findings indicate promising avenues for further exploration of LM4 analogs in human studies [25].

In our prospective study, we directly compared the lesion detection capabilities of [^68^Ga]Ga-DATA^5m^-LM4 and [^68^Ga]Ga-DOTANOC within a single patient cohort. Our findings strongly support the superiority of [^68^Ga]Ga-DATA^5m^-LM4, which is attributed to its enhanced ability to detect liver and skeletal metastases as well as its superior tumor-to-background ratio, resulting in improved image contrast.

The comparison of SULpeak and SULavg values between [^68^Ga]Ga-DATA^5m^-LM4 PET/CT and [^68^Ga]Ga-DOTANOC PET/CT revealed significant differences in uptake patterns across various physiological organs. Specifically, [^68^Ga]Ga-DATA^5m^-LM4 PET/CT demonstrated a lower uptake in several non-target organs compared to [^68^Ga]Ga-DOTANOC PET/CT, which could potentially impact diagnostic accuracy and specificity. In particular, the normal liver parenchyma, spleen, pituitary gland, kidney, blood pool, and salivary gland showed statistically significant lower uptake with [^68^Ga]Ga-DATA^5m^-LM4 PET/CT compared to [^68^Ga]Ga-DOTANOC PET/CT. These findings suggest that [^68^Ga]Ga-DATA^5m^-LM4 PET/CT may offer improved specificity by reducing non-specific uptake in these organs, thereby potentially enhancing lesion detection and characterization. However, it is noteworthy that the adrenal gland uptake did not show statistically significant differences between the two tracers, indicating similar uptake patterns in this organ. The observed differences in uptake patterns between the two tracers underscore the importance of considering the specific biodistribution and binding affinities of radiotracers in NET imaging. These findings highlight the potential utility of [^68^Ga]Ga-DATA^5m^-LM4 PET/CT in reducing non-target organ uptake, which may contribute to enhanced diagnostic accuracy and clinical utility in NET imaging. Further research is warranted to validate these observations and optimize imaging protocols for improved diagnostic performance.

The findings of this study reveal significant differences between [^68^Ga]Ga-DATA^5m^-LM4 PET/CT and [^68^Ga]Ga-DOTANOC PET/CT in detecting various types of lesions. Specifically, [^68^Ga]Ga-DATA^5m^-LM4 PET/CT demonstrated a higher detection rate for primary lesions, lymph nodes, liver metastases, and skeletal metastases compared to [^68^Ga]Ga-DOTANOC PET/CT. Notably, this difference was statistically significant for liver metastases and skeletal metastases, highlighting the superior performance of [^68^Ga]Ga-DATA^5m^-LM4 PET/CT in these sites. Moreover, when considering TBRs, [^68^Ga]Ga-DATA^5m^-LM4 PET/CT exhibited statistically significant higher values than [^68^Ga]Ga-DOTANOC PET/CT in primary lesions and liver metastases. These findings suggest a potentially greater sensitivity and accuracy of [^68^Ga]Ga-DATA^5m^-LM4 PET/CT in delineating tumor boundaries and identifying metastatic lesions, particularly in the liver. However, it is important to note that the differences in detection rates and TBR values were not statistically significant for lesions in other sites. This suggests that while [^68^Ga]Ga-DATA^5m^-LM4 PET/CT may offer advantages in certain anatomical locations, its superiority over [^68^Ga]Ga-DOTANOC PET/CT may not extend universally across all lesion types and sites.

No discernible uptake of the tracer was observed in any of the lung metastatic lesions, as evidenced by both [^68^Ga]Ga-DATA^5m^-LM4 PET/CT and [^68^Ga]Ga-DOTANOC PET/CT scans. This lack of uptake can be attributed to several factors. Firstly, the PET resolution utilized in this study was set at 6mm, which might have limited the ability to detect subtle uptake in smaller lesions such as those in the lungs. Additionally, active respiration induces continuous movement of the thorax, potentially obscuring tracer uptake in the lungs. It is plausible that enhancing the PET resolution could improve the detection of lung uptake in future studies. Hence, it is imperative to consider adjustments in PET resolution for subsequent investigations to better analyze tracer uptake patterns, particularly in regions prone to respiratory motion such as the lungs.

This uniform increase in SULpeak values with [^68^Ga]Ga-DATA^5m^-LM4 across different tumor grades could indicate a broader affinity of this tracer for a range of tumor grades and metastatic sites. Overall, this finding highlights the complexity of tracer behavior in molecular imaging and underscores the need for nuanced interpretation when comparing different radiotracers in clinical practice. Further research and larger-scale studies are warranted to fully understand the implications of these findings for diagnostic accuracy and treatment planning of NETs.

These findings emphasize the significance of choosing the most suitable tracer for particular clinical contexts, considering variables like lesion characteristics, tumor grade, anatomical site, and imaging preferences. It is essential to conduct further research and clinical validation to confirm these results and refine imaging approaches for enhanced cancer detection and characterization.

Our study findings align with recent research comparing SSTR antagonist PET/CT with SSTR agonist PET/CT. Lin et al. [16] conducted a study comparing [^68^Ga]Ga-NODAGA-JR11 and [^68^Ga]Ga-DOTATATE PET/CT in patients with metastatic, well-differentiated neuroendocrine tumors, demonstrating that [^68^Ga]Ga -NODAGA-JR11 exhibited superior sensitivity and a higher target-to-background ratio than [^68^Ga]Ga -DOTATATE. Similarly, Nicolas et al. [17] found that ^68^Ga-NODAGA-JR11 outperformed DOTATOC in terms of sensitivity, lesion detection, and image contrast in patients with low- or intermediate-grade gastroenteropancreatic NETs. Zhu et al. [18] reported that DOTA-JR11 showed superiority in patients with metastatic, well-differentiated neuroendocrine tumors, particularly in detecting liver metastases with a better tumor-to-background ratio, while ^68^Ga-DOTATATE may excel in detecting bone metastases.

Additionally, a phase I trial conducted by Reidy-Lagunes et al. [26] explored the use of [^177^Lu]Lu-radiolabeled SSTR2 antagonist, Satoreotide Tetraxetan, in heavily treated NETs, showing promising preliminary data for its efficacy. Moreover, in a proof-of-principle study conducted on a metastatic NET patient, [^177^Lu]Lu-AAZTA5-LM4 demonstrated sustained tumor uptake up to 72 h post-injection compared to a faster decline of background radioactivity. The patient also tolerated the treatment well [25]. Further investigations are necessary to assess the potential application of [^177^Lu]Lu-AAZTA5-LM4 in the treatment of SST2R-positive human NETs, building upon the promising outcomes of previous [^68^Ga]Ga-DATA^5m^-LM4 PET/CT studies.

### 3.2. Limitations of the Study

Although our study results prove that [^68^Ga]Ga-DATA^5m^-LM4 PET/CT is better than [^68^Ga]Ga-DOTANOC PET/CT in detecting a higher number of primary and metastatic lesions and providing a better TBR and better image contrast in physiological organs. There are a few limitations. One main limitation of this study is the use of CT as a reference standard (CECT in patients with a normal range of serum urea and serum creatinine) for identifying lesions (primary and metastatic) instead of using MRI; hence, this did not allow us to find the sensitivity and specificity accurately. Thus, in the future, we hope to use MRI as a reference standard. This study was performed with a sample size of only 50, which is considered to be small. Thus, in the future, this sample size can be increased. Another limitation is that no patients with brain metastases were included in the study, and hence, it was not possible to undertake the subgroup analysis of [^68^Ga]Ga-DATA^5m^-LM4 in the brain. Another limitation is that this is a single center study. The significant difference in the injected activities of the two tracers, as indicated by a p value of less than 0.001, also presents as a limitation of this study. While the difference in injected tracer activities may impact the SUV values, it does not directly impact detection rates. We strongly hope to address and mitigate these differences through careful standardization and data analysis strategies to minimize their impact on study outcomes.

### 3.3. Future Prospects

It can be noted that the number of skeletal and liver metastatic lesions are higher and more statistically significant in [^68^Ga]Ga-DATA^5m^-LM4 PET/CT than [^68^Ga]Ga-DOTANOC PET/CT. Thus, it can be stated that [^68^Ga]Ga-DATA^5m^-LM4 PET/CT can be used to quantify the extent of skeletal and liver metastases for better planning of SSTR agonist- or antagonist-based therapy. In the future, a study with [^68^Ga]Ga-DATA^5m^-LM4 PET/CT in comparison with SSTR agonists can be undertaken in NET patients with a large sample size, and more studies on therapeutic action based on the DATA^5m^-LM4 ligand can be performed. And a detailed comparison between [^68^Ga]Ga-DATA^5m^-LM4 and [^68^Ga]Ga-DOTANOC PET/CT in a subgroup analysis according to the grade of tumor can be conducted.

## 4. Materials and Methods

### 4.1. Inclusion Criteria and Exclusion Criteria

All patients who provided consent for the study were consecutively enrolled based on the following eligibility criteria: histologically confirmed neuroendocrine tumors, cases with positive or inconclusive findings in [^68^Ga]Ga-DOTANOC PET/CT, patients off long-acting or short-acting octreotide for a minimum of 4 weeks and 24 h, respectively, patients willing and able to undergo imaging at the specified time-points outlined in the protocol, and patients who underwent both [^68^Ga]Ga-DOTANOC and [^68^Ga]Ga-DATA^5m^-LM4 PET/CT within a 2 week interval. Exclusion criteria comprised lactating or pregnant females, patients unwilling to provide informed consent, individuals with known bladder outlet obstruction, concurrent severe and/or uncontrolled and/or unstable medical conditions, such as ischemic heart or lung disease, posing unacceptable risks, and any condition judged by the investigator to impede compliance with protocol requirements. Based on these eligibility criteria, 50 patients were included in the study.

### 4.2. Synthesis of [^68^Ga]Ga-DATA^5m^-LM4 and Quality Control

The DATA^5m^-LM4 precursor was sourced from the Department of Chemistry—TRIGA, Johannes Gutenberg University, Mainz, Germany. Gallium-68 was extracted from a ^68^Ge/^68^Ga generator using 0.1 M HCl, and its pH was adjusted by mixing it with a sodium acetate buffer. The DATA^5m^-LM4 precursor was added to a sodium acetate buffer with a pH of 5.0. This mixture was then combined with the eluate from the generator. Radiochemical yields (RCY) ranging from 80% to 95% were achieved within 10 min at 90 °C. Subsequently, purification, formulation, and sterile filtration processes were carried out. Quality control measures were performed using both analytic high-performance liquid chromatography and thin-layer chromatography, both showing radiochemical purities (RCP) exceeding 95%. The same procedure was followed for DOTANOC. Three patients were injected from each batch.

### 4.3. PET/CT Acquisition

The average injection doses for [^68^Ga]Ga-DATA^5m^-LM4 and [^68^Ga]Ga-DOTANOC were 4.1 mCi (150 MBq) (25th to 75th percentile: 2.8 to 5.6 mCi) and 2.5 mCi (92.5 MBq) (25th to 75th percentile: 2.2 to 3.0 mCi), respectively.

Imaging was performed 50–60 min after the intravenous administration of both radiotracers, with the patient in a supine position for all acquisitions. The imaging protocol involved acquiring a CT and a PET scan following an initial scout image to define the field of view. Reconstruction parameters were set as follows: reconstruction method “VUE point FX” (Iterative reconstruction), quantitation method “SharpIR”, *Z*-axis filter “Standard”, filter cut-off (mm) “6.4”, subsets “24”, and iterations “2”. Scans were conducted using a 128-slice GE Discovery 710 PET/CT scanner (Manufacturer—GE Healthcare, Chicago, IL, USA) with a 40 mm detector rotating at a speed of 0.35 s. The PET scan duration was 2 min per bed, covering approximately 10 beds from head to mid-thigh for each patient.

A diagnostic dose CT scan was conducted with parameters set to 300 to 380 mAs at 120 kVp, a slice thickness of 3.75 mm, and a pitch of 0.6. The image processing and analysis were performed using a GE Xeleris workstation. Qualitative and quantitative comparisons of the tracers were conducted for both the [^68^Ga]Ga-DOTANOC and [^68^Ga]Ga-DATA^5m^-LM4 PET/CT scans.

Following the completion of the head-to-mid-thigh scan, a PET/CT spot image covering the abdomen and pelvis was obtained. Contrast-enhanced PET/CT scans were performed in patients meeting the IV contrast eligibility criteria, utilizing a non-ionic isomolar contrast medium (Iodixanol injection, USP) administered intravenously at a dosage of 1 mL/kg body weight, containing 320 mg/mL as specified by the principal investigator. The CT parameters for this procedure included 120 to 380 mA at 120 kVp, and the acquisition of slices was at a thickness of 1.5 mm and a pitch of 0.6.

Patients eligible for this imaging protocol exhibited laboratory results within the specified reference range at the screening visit, with serum urea levels between 17 and 49 mg/dL and serum creatinine levels between 0.7 and 1.2 mg/dL. Additionally, these patients had no history of contrast allergic reactions. Oral contrast was administered to individuals with confirmed or suspected gastric or enteric sites of primary metastases.

### 4.4. Data Interpretation

To evaluate the diagnostic efficacy of both radiotracers, patient-based and lesion-based analyses were performed for primary and metastatic lesions. Additionally, a tumor-to-background ratio analysis of physiological organs was conducted. Two nuclear medicine physicians independently interpreted the PET/CT scan results for both [^68^Ga]Ga-DATA^5m^-LM4 and [^68^Ga]Ga-DOTANOC. They were blinded to patients’ clinical history and histopathological (HPE) status. Any discrepancies in interpretation were resolved through consensus discussions.

### 4.5. Data Analysis and Processing

The qualitative analysis involved visually assessing radiotracer uptake, which was corroborated by morphological findings using CT as the reference standard. Lesions in the liver were considered positive if they appeared hypodense on a non-contrast CT and hyperenhanced after intravenous contrast administration, adhering to the eligibility criteria for contrast use. These lesions were required to be rounded with a minimum size of 1 cm, with or without necrosis. Primary lesions were evaluated using the same enhancement criteria, without minimum size or shape requirements. In the case of skeletal lesions, both lytic and sclerotic lesions on CT were deemed positive. For lymph nodes, those with a rounded or oblique shape and a size of 1 cm or more were considered positive. Lesion counts on CT served as the reference standard for quantitative analysis.

When radiopharmaceutical uptake exceeded background levels, a lesion was considered positive on PET. The uptake in the lesions on both scans was compared to the morphological features of the CT counterpart. A 3D auto-contour ROI at a 30% SULpeak and SULavg threshold was meticulously delineated around the site of [^68^Ga]Ga-DOTANOC and [^68^Ga]Ga-DATA^5m^-LM4, expressing lesions on transaxial images for quantitative comparisons. To quantitatively compare the uptake in the lesions between the radiotracers, the ROIs were presented as standardized uptake values (SUV) corrected for lean body mass: SULpeak and SULavg. The ROI measurement was a 3 cm spherical ROI in the normal right lobe of the liver (for liver), a 1.2 cm ROI within the descending aorta (blood pool), and 1 cm for other physiological organs. The SUV values corrected for lean body mass (peak and average) for both [^68^Ga]Ga-DOTANOC and [^68^Ga]Ga-DATA^5m^-LM4 were recorded for each site. The tumor-to-background ratio (TBR) was determined by dividing the standardized uptake value at peak (SULpeak) and the standardized uptake value at average (SULavg) of the primary tumor or metastases by the corresponding values of background SULpeak and SULavg. In the case of bilateral organs, such as the adrenal glands and renal cortex, the average SULpeak and SULavg were calculated.

### 4.6. Definitions

True-positive (TP) lesion: uptake in the lesion seen on [^68^Ga]Ga-DOTANOC/[^68^Ga]Ga-DATA^5m^-LM4 images were higher than the background and were found to be positive on diagnostic CT/histological examination.

False-positive (FP) lesion: uptake in the lesion seen on [^68^Ga]Ga-DOTANOC/[^68^Ga]Ga-DATA^5m^-LM4 images were higher than the background and were found to be negative on diagnostic CT/histological examination.

True-negative (TN) lesion: no uptake seen on [^68^Ga]Ga-DOTANOC/[^68^Ga]Ga-DATA^5m^-LM4 PET/CT images were higher than the background, and the results were found to be negative on diagnostic CT/histological examination.

False-negative (FN) lesion: lesions that were missed in [^68^Ga]Ga-DOTANOC/[^68^Ga]Ga-DATA^5m^-LM4 images and were higher than the background were found to be positive for malignancy at diagnostic CT/histological examination.

Background: the uptake value measured at the right lobe of the normal area of the liver with a spherical ROI of 3 cm was considered as the background uptake value.

### 4.7. Adverse Event Monitoring

Vital signs (blood pressure and heart rate) and clinical symptoms were monitored and recorded for up to 2 h after injection. Adverse events were recorded according to version 4.03 of the Common Terminology Criteria for Adverse Events.

### 4.8. Statistical Analysis

The statistical analysis was performed using MedCalc statistical software (v15.0). Continuous variables were presented in terms of mean, median, standard deviation (SD), range, and interquartile range (IQR). Since it is a two-grouped paired data, which is not normally distributed, [^68^Ga]Ga-DOTANOC and [^68^Ga]Ga-DATA^5m^-LM4 uptakes were compared using a Wilcoxan signed-rank test. The sensitivities of [^68^Ga]Ga-DOTANOC and [^68^Ga]Ga-DATA^5m^-LM4 PET/CT examinations were calculated and compared. *p* values ≤ 0.05 were considered significant. Indeterminate results from the [^68^Ga]Ga-DATA^5m^-LM4 and [^68^Ga]Ga-DOTANOC PET/CT tests were approached as false-negative or false-positive using a diagnostic quality CT scan as the reference standard. Participants with missing data including histopathology, tests, or reference standard imaging were excluded from the analysis.

## 5. Conclusions

While the [^68^Ga]Ga-DATA^5m^-LM4 SSTR antagonist, when compared to the [^68^Ga]Ga-DOTANOC PET SSTR agonist, demonstrated nearly equivalent sensitivity for primary lesions and lymph nodal metastases, the new antagonist exhibited superior detection efficiency in imaging distant metastatic lesions. [^68^Ga]Ga-DATA^5m^-LM4 showed improved sensitivity and a higher target-to-background ratio compared to [^68^Ga]Ga-DOTANOC PET/CT. Considering the increased TBR for [^68^Ga]Ga-DATA^5m^-LM4, it is anticipated that target lesions will receive adequate radiation, while non-target lesions will receive less. For patients whose disease has progressed after undergoing treatment with agonist therapy, treatment can be pursued with antagonists (based on DATA^5m^-LM4). We strongly believe that [^68^Ga]Ga-DATA^5m^-LM4 can be utilized in the future for the comprehensive evaluation of distant metastases to facilitate accurate treatment planning.

## Figures and Tables

**Table 1:** Patient demographics.

Characteristic	Value (%)
**Age (mean ± SD, range)**	mean ± SD: 44.7 ± 13.7, range: 14 to 71
**Sex**	
Male	24 (48%)
Female	26 (52%)
**Primary tumor site**	
Pancreas	23 (46%)
Gastric	6 (12%)
Duodenal	4 (8%)
Gastro-enteric	2 (4%)
Gall bladder	1 (2%)
Jejunum	3 (6%)
Rectum	1 (1%)
Ileum	3 (6%)
Small intestine	1 (2%)
Unknown primary	6 (12%)
**CgA (median, IQR) (ng/mL)**	201 (94 to 812)
**Extent of metastases**	
**Liver**	31 (62%)
**Lymph nodes**	
Head and neck	4 (8%)
Thoracic	5 (10%)
Abdomino-pelvic	27 (54%)
Total lymph nodal	8 (16%)
**Bone**	6 (12%)
**Lung**	8 (16%)
**Other sites**	0
**WHO tumor grade**	
Grade I	23 (46%)
Grade II	16 (32%)
Grade III	6 (12%)
Unknown grade	5 (10%)

**Table 2:** Comparison of normal organ uptake values.

Organ	SULpeak			SULavg		
	DATA^5m^ -LM4	DOTANOC	*p*-Value	DATA^5m^-LM4	DOTANOC	*p*-Value
Pituitary gland	2.1	1.7	0.025	2.7	2.0	0.540
	(1.2 to 3.5)	(0.8 to 2.5)		(1.2 to 4.9)	(0.9 to 3.9)	
Parotid gland	1.2	0.6	<0.0001	0.9	0.6	0.0005
	(0.7 to 1.5)	(0.4 to 1.1)		(0.6 to 1.3)	(0.3 to 1.0)	
Blood pool (descending aorta)	0.8	0.4	0.0003	0.7	0.4	0.0004
	(0.4 to 1.1)	(0.3 to 0.7)		(0.4 to 0.9)	(0.3 to 0.7)	
Liver	2.5	3.5	0.01	2.0	2.7	0.015
	(1.7 to 3.8)	(2.4 to 5.3)		(1.3 to 3.1)	(1.9 to 4.0)	
Spleen	6.6	11.3	<0.0001	5.7	10.3	<0.0001
	(4.0 to 9.7)	(7.2 to 18.3)		(3.7 to 8.6)	(5.8 to 13.9)	
Kidney	9.8	6.1	0.0004	8.9	4.2	<0.0001
	(6.6 to 13.8)	(4.1 to 9.6)		(5.9 to 11.5)	(2.9 to 6.8)	
Adrenal glands	4.1	5.4	0.695	3.4	4.4	0.556
	(2.4 to 6.1)	(1.6 to 6.6)		(1.9 to 5.1)	(1.1 to 5.5)	

Data are expressed as median (IQR).

**Table 3:** Comparison of various parameters between [^68^Ga]Ga-DOTANOC and [^68^Ga]Ga-DATA^5m^-LM4 PET/CT according to the site of disease in 50 patients.

Parameters	Imaging Method	Primary	Lymph Node Metastasis	Lung Metastasis	Liver Metastasis	Bone Metastasis
Patient-based analysis						
	CT	33	31	8	31	6
	[^68^Ga]Ga-DOTANOC	28 (84.8%)	23 (74.1%)	0 (0%)	26 (83.9%)	6 (100%)
	[^68^Ga]Ga-DATA^5m^-LM4	28 (84.8%)	23 (74.1%)	0 (0%)	28 (90.3%)	6 (100%)
	*p*-value	1.000	1.000	-	0.449	1.000
Lesion-based analysis						
	CT	47	107	12	283	41
	[^68^Ga]Ga-DOTANOC	40 (85.1%)	82 (76.6%)	0 (0%)	253 (89.4%)	34 (82.9%)
	[^68^Ga]Ga-DATA^5m^-LM4	42 (89.4%)	83 (77.6%)	0 (0%)	292 (value greater than CT) = 100%	45 (value greater than CT) = 100%
	*p*-value	0.544	0.862	-	<0.0001	0.005
SULmean						
	[^68^Ga]Ga-DOTANOC	8.0 (4.5 to 13.9)	5.8 (2.2 to 17.9)	-	8.8 (4.8 to 17.9)	1.0 (0.6 to 3.6)
	[^68^Ga]Ga-DATA^5m^-LM4	8.58 (4.99 to 12.68)	6.1 (2.1 to 12.4)	-	9.3 (6.3 to 15.7)	3.7 (2.2 to 5.5)
	*p*-value	0.981	0.935	-	0.750	0.312
SULpeak						
	[^68^Ga]Ga-DOTANOC	13.4 (6.1 to 19.0)	5.6 (3.3 to 20.5)	-	14.7 (7.3 to 20.9)	1.1 (1.0 to 1.6)
	[^68^Ga]Ga-DATA^5m^-LM4	13.6 (6.6 to 20.2)	6.7 (3.9 to 19.3)	-	15.7 (8.3 to 25.7)	3.0 (2.3 to 4.5)
	*p*-value	0.316	0.708	-	0.484	0.312
Tumor-to-liver ratios SULpeak						
	[^68^Ga]Ga-DOTANOC	3.0 (2.0 to 5.6)	2.6 (0.7 to 5.3)	-	4.1 (1.9 to 6.4)	0.8 (0.3 to 1.6)
	[^68^Ga]Ga-DATA^5m^-LM4	4.3 (3.1 to 8.4)	3.1 (1.5 to 6.1)	-	7.7 (3.3 to 14.4)	2.0 (0.6 to 2.8)
	*p*-value	0.014	0.757	-	0.008	0.093
Tumor-to-liver ratios SULmean						
	[^68^Ga]Ga-DOTANOC	3.3 (2.1. to 5.7)	3.5 (1.3 to 5.7)	-	3.9 (1.6 to 6.8)	0.9 (0.4 to 2.2)
	[^68^Ga]Ga-DATA^5m^-LM4	3.8 (2.8 to 6.7)	3.4 (1.6 to 5.0)	-	4.2 (2.6 to 8.1)	2.4 (2.1 to 3.2)
	*p*-value	0.034	0.961	-	0.106	0.093

Data are expressed as median (IQR) for SULmean, SULpeak, and tumor-to liver ratios.

## Data Availability

Data available on request due to restrictions.

## Appendix A

**Table A1:** Detailed patient clinical parameters.

						Site of Metastases						
S. No	Sex	Age	Primary Site of Tumor	Tumor Grade	Ki-67 Index	Liver	Lymph Node	Bone	Others	Tumor Marker (CgA)	IHC Markers	Prior Treatments
1.	M	38	Pancreas	II	<3	yes	Yes	no	no	109	Synaptophysin	Nil
2.	F	50	Pancreas	N/A	-	yes	Yes	yes	no	488	Synaptophysin, NSE	Chemotherapy, 7 cycles
3.	M	41	Pancreas	I	<3	yes	Yes	no	no	165.89	Synaptophysin, CgA	Nil
4.	M	64	Ileum	I	<3	no	Yes	no	no	597	Not performed	Nil
5.	F	44	Pancreas	II	3–20	yes	no	yes	no	23.74	Synaptophysin, CgA	pancreatio-spleenectomy
6.	F	66	Pancreas	II	3–20	yes	Yes	no	no	2282	Cytokeratin, Synaptophysin, CgA	Nil
7.	M	50	Pancreas	I	<3	no	Yes	no	no	254.9	Synaptophysin, CgA	Nil
8.	M	30	Gastric	I	<3	yes	Yes	Yes	no	1278	Synaptophysin	Nil
9.	M	50	Jejunum	II	3–20	no	no	no	no	67.09	Not performed	Liver metastatectomy
10.	F	25	Small intestine	Poorly differentiated NET	-	yes	Yes	no	Yes	233.5	Not performed	Surgery and metastatectomy,5 cycles of [177Lu]Lu-PRRT
11.	M	41	Duodenal	I	<3	no	Yes	no	no	22.4	Synaptophysin, CgA	Gastrojejunostomy, Octreotide
12.	M	62	Jejunum	II	3–20	yes	Yes	no	no	17.23	Synaptophysin, CgA, CD56, PCK.	Octreotide
13.	M	62	Duodenal	I	-	no	Yes	no	no		Not performed	Nil
14.	M	67	Pancreas	I	<3	yes	no	no	no	834	Pancytokeratin, CgA	6 cycles of [177Lu]Lu-PRRT
15.	F	58	Pancreas	I	<3	yes	Yes	no	no	1042	Not performed	Octreotide
16.	F	64	Gastric	II	3–20	yes	no	no	no	1500	Synaptophysin, CgA, CD56, cytokeratin.	Nil
17.	F	44	Gastro-enteric	III	>20	no	no	no	no		Not performed	One cycle of capecitabine/temozolomide (CAPTEM)
18.	F	29	Pancreas	III	>20	no	no	no	no	344.4	CgA, Synaptophysin.	Nil
19.	M	58	Rectum	III	-	yes	Yes	yes	no		Cytokeratin, Synaptophysin, CgA	Nil
20.	M	47	Pancreas	Poorly differentiated NET	-	yes	Yes	no	no		Not performed	6 cycle of chemotherapy
21.	F	48	Ileum	I	<3	yes	Yes	no	no	358.5	Not performed	Right hemicolectomy
22.	M	54	Pancreas	I	<3	no	no	no	no	430.4	Synaptophysin, CgA, INSM-1	Nil
23.	F	57	Pancreas	I	<3	no	no	no	no	950	Synaptophysin, CgA	Pancreatectomy and gastrojejunostomy
24.	M	45	Unknown primary	I	<3	no	Yes	no	no	153.3	Synaptophysin, CgA, PCK.	Nil
25.	F	44	Pancreas	II	3–20	yes	no	no	no	156	Not performed	Whipples, post bilateral inferior parathyroidectomy, truncal vagotomy
26.	F	42	Pancreas	II	3–20	no	no	no	no		CK, Synaptophysin, CgA, CD 56.	Nil
27.	F	35	Duodenal	I	<3	no	Yes	no	no	113	Synaptophysin, chromogranin.	Post surgery
28.	M	38	Gastro-enteric	II	3–20	yes	Yes	no	no	1500	Not performed	Nil
29.	F	30	Pancreas	II	3–20	no	no	no	no	28.62	Cytokeratin, Synaptophysin, CgA	Surgical excision
30.	M	43	Unknown primary	I	<3	yes	no	no	no		Synaptophysin, CgA	Nil
31.	F	55	Ileum	III	>20	yes	Yes	no	no		Synaptophysin, CgA, CD56.	Inj. Octreotide
32.	F	14	Unknown primary	III	>20	yes	no	no	no		CgA, pancytokeratin and snaptophysin	Nil
33.	F	36	Gastric	II	3–20	no	no	no	no	68.06	CD 56 and synaptophysin	Gastric wide local excision
34.	M	40	Gastric	N/A		yes	Yes	no	no	789.8	Synaptophysin, CgA.	Nil
35.	F	41	Pancreas	II	3–20	no	no	no	no		Not performed	Nil
36.	F	49	Unknown primary	II	3–20	yes	Yes	no	no		Not performed	Radical hysterectomy
37.	M	15	Pancreas	N/A		no	Yes	no	no		Not performed	Nil
38.	M	44	Pancreas	I	<3	yes	Yes	yes	no	12	Synaptophysin and CgA	Octreotide, everolimus
39.	M	34	Gastric	I	<3	no	no	no	no		Cytokeratin, Synaptophysin, CgA	Nil
40.	M	42	Pancreas	I	<3	yes	Yes	no	no	165.89	Synaptophysin, CgA	Octreotide, everolimus
41.	M	40	Jejunum	I	<3	no	Yes	no	no	117.98	CgA	Nil
42.	F	60	Pancreas	I	<3	yes	Yes	no	no		CgA, synaptophysin	Octreotide
43.	F	37	Gall bladder	III	>20	yes	Yes	yes	no		CgA and CK 19	6 cycles of chemotherapy
44.	F	21	Unknown primary	II	3–20	no	Yes	no	no	1846.11	Cytokeratin, INSM 1 and CgA	Nil
45.	F	56	Duodenal	I	<3	Yes	Yes	no	no		CgA and synaptophysin	Octreotide
46.	F	44	Pancreas	II	3–20	Yes	Yes	no	no	130	CgA and synaptophysin	Octreotide
47.	M	27	Gastric	I	<3	No	no	no	no		Synaptophysin, NSE, S100, CD56.	Exploratory laparotomy, repair of perforation site, and feeding jejunostomy for perforation peritonitis.
48.	M	71	Unknown primary	II	3–20	Yes	Yes	no	no		CgA and synaptophysin	Nil
49.	F	62	Pancreas	I	<3	yes	no	no	no	201	Synaptophysin, CgA and INSM1	6 cycles of Lu-PRRT
50.	F	24	Pancreas	I	<3	yes	no	no	no	79.5	CgA and synaptophysin	Pancreatectomy, splenectomy, octreotide

Male is expressed as M and Female is expressed as F in sex column; CgA: chromogranin A; PRRT: peptide receptor radionuclide therapy; N/A: Not available.
